# Complete chloroplast genomes analysis of *Lamium barbatum* and *Leucas ciliata* (Lamiaceae)

**DOI:** 10.1080/23802359.2025.2582528

**Published:** 2025-11-09

**Authors:** Qing Du, Baoxiang Xue, Benyi Tan

**Affiliations:** ^a^ College of Pharmacy, Key Laboratory of High-value Utilization of Characteristic Economic Plants, Key Laboratory of Plant Chemistry on Qinghai Tibet Plateau, Qinghai Minzu University, Xining, China; ^b^ Fresh Sky (Beijing) International Technology Co., Ltd., Beijing, China; ^c^ College of Traditional Chinese Pharmacy, Jilin Agricultural Science and Technology University, Jilin City, China; ^d^ Tibet Rhodiola Pharmaceutical Holding Co., Ltd., Tibet Pharmaceuticals Traditional Chinese Medicine Planting Base, Linzhi City, China

**Keywords:** *Lamium barbatum*, *Leucas ciliata*, chloroplast genome, SSR analysis, phylogenetic relationship

## Abstract

The two Lamioideae species, *Lamium barbatum* Siebold & Zucc. 1846, is distributed across Europe, North Africa, Asia, and North America, while *Leucas ciliata* Hochst. ex Benth. 1829 is found in southern China and Southeast Asia. We sequenced their complete chloroplast genomes using the Illumina platform, obtaining lengths of 150,887 bp and 151,259 bp, respectively. Phylogenetic analysis showed *Lamium barbatum* is closely related to four other *Lamium* species, including *Lamium takeshimense*, *Lamium album*, *Lamium amplexicaule*, and *Lamium galeobdolon*. The *Leucas ciliata* species clusters with *Leucas mollissima*. These findings provide valuable data for species identification, evolutionary research, and conservation genetics.

## Introduction

The Lamiaceae is the sixth-largest angiosperm family with worldwide distribution. Most genera are predominantly distributed in Asia, Africa, and Europe, with approximately 1387 species within 100 genera in China (Chang et al. 2014). Many species possess significant economic, ornamental, aromatic, and medicinal value, as well as the ability to activate blood circulation (Guzman and Molina 2018). The two species of *Lamium album* subsp. *barbatum* Siebold & Zucc. 1846 and *Leucas ciliata* Hochst. ex Benth. 1829 in this study belong to different genera within the Lamioideae subfamily of the Lamiaceae family, as classified by chloroplast regions (Cantino and Sanders 1986; Bendiksby et al. 2011). Previous studies have shown that *Lamium album* subsp. *barbatum* contains various bioactive compounds, including flavonoid glycosides, phenylpropanoid glycosides, sterols, steroids, and cycloartane glycosides, which can be used for the treatment of traumatic injuries, pediatric malnutrition, and diseases affecting the uterine and urinary systems (Tjendana Tjhin et al. 2024). As species of the same subfamily, *Leucas ciliata* and *Anisomeles indica* are two varieties of ‘Fang feng’ in the Lamiaceae family, including differentiated main chemicals (Gui et al. 1991). The species *Leucas ciliata* contains tricin, cirsilineol, daucosterol, leucasin, and 17 phenolic acid compounds, which have effects on regulating liver blood flow, removing wind, and detoxifying (Wang et al. 2012; Kuang et al. 2024).

The chloroplast genome and its key genes, due to their highly conserved structure and moderate mutation rate, play a crucial role in the biosynthesis and metabolism of plant chemical components, particularly in the phylogenetic development of biodiversity species (Wang et al. 2024). By regulating photosynthetic rates across plant species, chloroplasts significantly influence growth efficiency, reproductive success, and breeding outcomes (Zhang et al. 2023). These organelles not only act as energy production hubs but also drive carbon assimilation processes, fundamentally shaping ecosystem energy flows. Crucially, chloroplasts are deeply intertwined with plant metabolism. Many bioactive compounds in traditional medicinal plants originate from secondary metabolites synthesized within chloroplasts, demonstrating a close evolutionary connection between chloroplast evolution and their medicinal value (Gomez-Casati et al. 2022). The chloroplast genomes from 288 species in 191 genera of the Lamiaceae family were used to construct a phylogenetic relationship, which revealed new findings indicating that these species form twelve supported, updated clades (Li et al. 2016). Many species of the subfamily and genera of Lamioideae have been studied in terms of their chloroplast genome and molecular systematics, including *Lamium takeshimense*, *Thymus mongolicus*, *Lycopus lucidus*, and *Mentha canadensis* (Huaizhu et al. 2019, 2020; Park et al. 2019; Wang et al. 2021). Whereas the complete chloroplast genomes of the two species in this study have not been reported, nor have their morphological and genomic characteristics been compared. Therefore, this study addresses these gaps to explore the analysis of chloroplast genomes and their evolutionary relationships, gaining a better understanding of these species.

## Materials and methods

**Figure 1.**
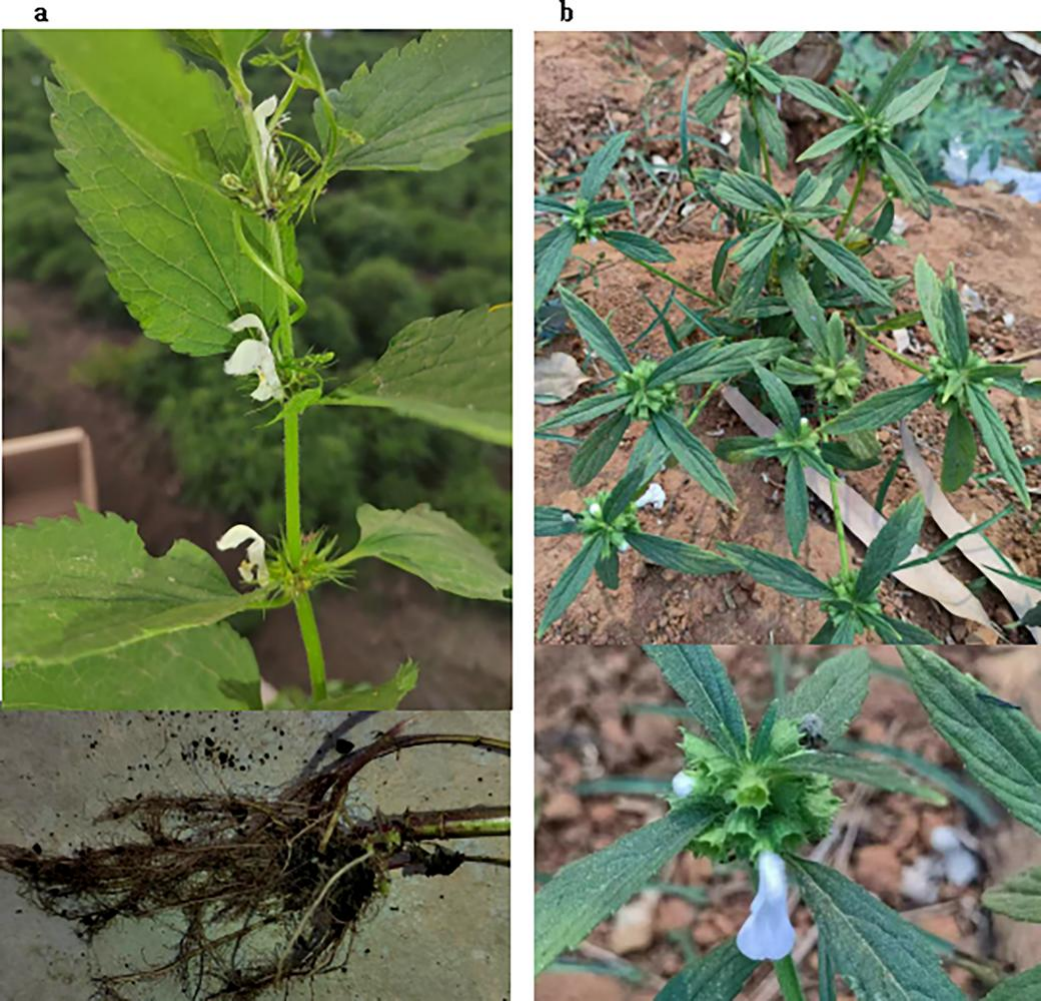
Morphological characteristics of the Lamium barbatum (a) and Leucas ciliata (b).

Fresh leaves of *Lamium barbatum* and *Leucas ciliata* (Figure 1) were collected from Zuojia County (44°3′18″ N, 126°5′54″ E), Jilin City of Jilin Province, China, and Xuwen County (20°16′48ʺ N, 110°11′24ʺ E), Zhanjiang City of Guangdong Province, China. The specimens were deposited at the National Plant Specimen Resource Center (Li Zexin, pelizexin@ibcas.ac.cn) and Specimen Museum, Institute of Botany, Chinese Academy of Sciences, Jiangsu Province (Dong Xiaoyu, nas@cnbg.net), under the voucher numbers Duq2023013 and Duq2022080. The two plants were identified by Dr. Linqinwen, Dr. Liubing, and Dr. Limin (Institute of Botany, Chinese Academy of Sciences, linqinwen83@163.com and liubing@ibcas.ac.cn), Mr. Louanrui (Bozhou Planting Agriculture Technology Co., Ltd., Bozhou, China, 2281384216@qq.com), and the authors.

*Characteristics*: (a) herbs, perennial, rhizome with long underground creeping branches; stem is solitary, erect, quadrangular, hollow, and nearly hairless; the leaf is ovate or reniform to obovate; the calyx is bell-shaped, and the corolla is white or light yellow. (b) Herbs, annual, the stem is erect or twisted at the top, densely covered with golden yellow long and hard hairs; the leaves are ovate-lanceolate or lanceolate, and the corolla is white or purple with slightly soft hairs on the outside near the throat. The photographs were taken by Xue Baoxiang on 2 June 2023 (a) and by Lou Anrui on 13 April 2023 (b).

The total genomic DNA of the two species was extracted using the Plant Genomic DNA Kit (Tiangen Biotech, Beijing, China). The quality detection of DNA purity and concentration was executed in a DNA library at the laboratory of Genepioneer Biotechnology Co., Ltd. (Nanjing, China). The qualified samples were then sequenced on an Illumina HiSeq 3000 platform (San Diego, CA). The raw data were filtered (Q30 is greater than 85%), assembled, annotated, corrected, and visualized using GetOrganelle v1.7.5 and CPGview software as described in the reference, as well as the analysis of simple sequence repeats (SSRs) (http://www.1kmpg.cn/cpgview, Jin et al. 2020; Du et al. 2022; Liu et al. 2023). The sequencing depth has been aligned, acquired, treated, and represented using CPtools, Bowtie2, and ggplot2 software (Langmead and Salzberg 2012; Wickham 2016; Huang et al. 2024). After that, the annotated sequences from the two chloroplast genomes were submitted to the GenBank database of the National Center for Biotechnology Information (NCBI) to obtain the accession numbers PV802395.1 (*Lamium barbatum*) and PV802396.1 (*Leucas ciliata*).

The phylogenetic analysis was constructed based on complete chloroplast genomes of the two species and 17 other species downloaded from the NCBI database using the Phylosuite software (version 1.2.3, http://phylosuite.jushengwu.com/, Xiang et al. 2023). The species *Lithospermum erythrorhizon* served as the outgroup species. The phylogenetic method of construction and illustration referred to the publication (Yuan et al. 2024) with 1000 replications as the assessing bootstrap standard.

## Results

**Figure 2.**
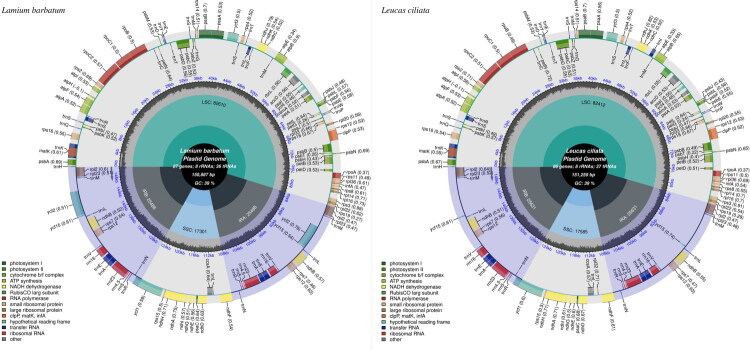
Graphic representation in the chloroplast genomes of the Lamium barbatum (a, PV802395.1) and Leucas ciliata (b, PV802396.1) using CPGView (http://www.1kmpg.cn/cpgview).

The length of the chloroplast genome of *Lamium barbatum* and *Leucas ciliata* is 150,887 bp and 151,259 bp, with the typical quadripartite structure (Figure S1), comprising a large single-copy region (LSC, 82,610 bp and 82,412 bp), a small single-copy region (SSC, 17,301 bp and 17,585 bp), and two inverted repeat regions (IR, 25,488 bp and 25,631 bp by each) (Figure 2, Table S1). The GC contents of the two total chloroplast genomes are 38.5% and 38.6% and that of IR regions (43.39% and 43.41%) is higher than LSC regions (36.84% and 36.87%) and SSC regions (32.76% and 32.55%). After mapping and calculation, the coverage ranges of the sequencing data were all 99.86%. The sequencing depth range of genomes varied from 33× to 3830× (*Lamium barbatum*) and from 26× to 7046× (*Leucas ciliata*) (Figure S2). A total of 131 genes were identified within the two species, including 87 (*Lamium barbatum*) and 86 (*Leucas ciliata*) protein-coding genes, 36 (*Lamium barbatum*) and 37 (*Leucas ciliata*) tRNA genes, and eight rRNA genes (Table S1). Seven protein-coding genes are duplicated in the IR regions, including *rps*7, *rps*12, *rpl*2, *rpl*23, *ndh*B, *ycf*2, and *ycf*15 (Table S2). Within the two studied chloroplast genomes, cis-splicing was detected in twelve genes, and two trans-splicing *rps*12 genes were also identified at diverse locations (Figures S3 and S4 and Table S3), which included two introns and three exons, in addition to the other two cis-splicing genes, *ycf*3 and *clp*P.

Each map contains seven circles, each representing the distribution of repeats, microsatellite sequences, the size of the four regions, GC content, and genes (Du et al. 2022).

In this study, a total of 22 and 26 SSRs with the length range of 10–41 bp and 10–87 bp were detected in the two chloroplast genomes of *Lamium barbatum* and *Leucas ciliata* (Table S4), among which were two types of repeats, that is, most of the mono-nucleotide repeats (18 for *Lamium album* subsp. *barbatum* and 20 for *Leucas ciliata*), and the dinucleotide repeats (three for *Lamium barbatum* and four for *Leucas ciliata*). The number of complex repeats within the two species is one and two by each species (Table S5). The SSR sequences of each species can be amplified by designing primers at both ends through PCR to obtain the target DNA sequences. The size of the amplification products is then analyzed by gel electrophoresis to determine the genotype of each sample.

**Figure 3.**
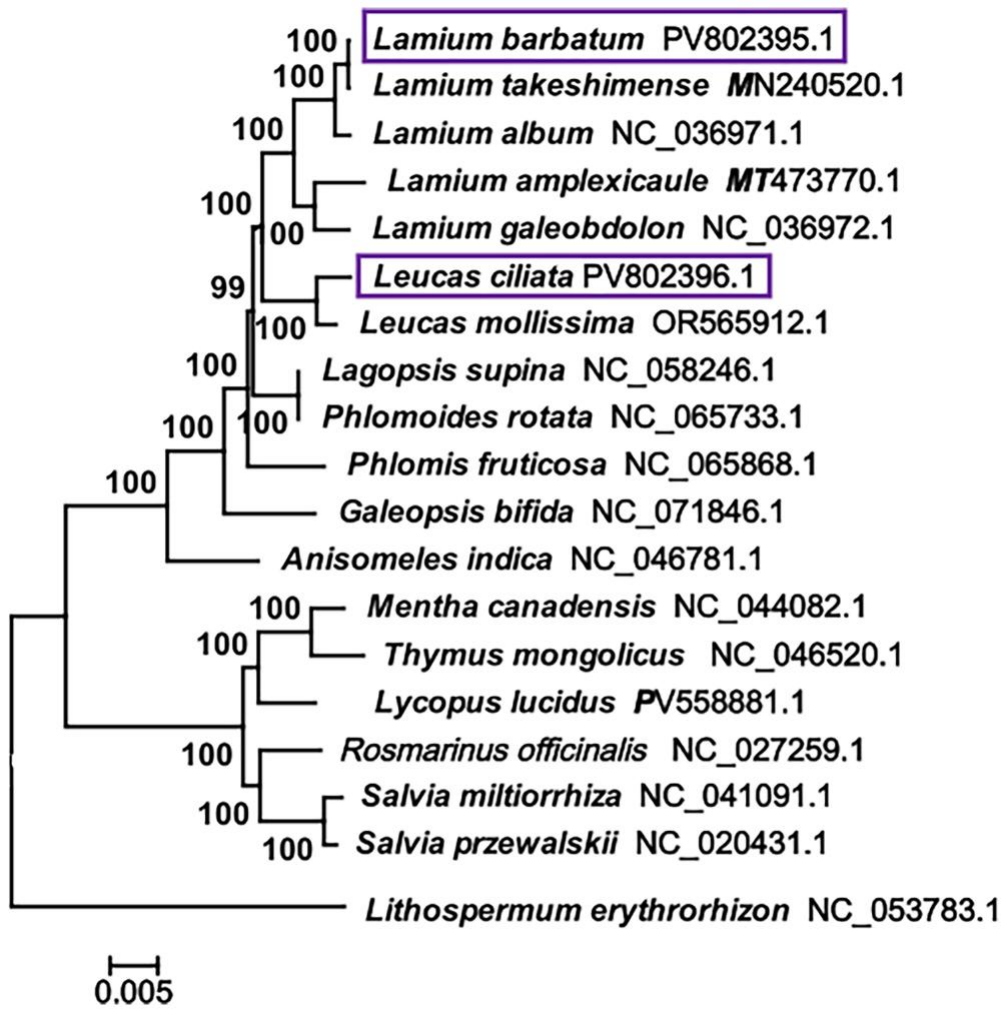
The maximum-likelihood (ML) phylogenetic tree in the 19 studied species based on the nucleotide sequences of 68 shared CDS genes within the complete genomes.

The results revealed that the two species clustered into a single larger clade, whereas they were located at different branches, with closely related species in the same genus (Figure 3). Within this framework, *Lamium barbatum* was found to be most closely related to the four *Lamium* species, including *Lamium takeshimense*, *Lamium album*, *Lamium amplexicaule*, and *Lamium galeobdolon.* The species *Leucas ciliata* was grouped with *Leucas mollissima* into a single clade. All the evolutionary clades were well-supported, united groups with high bootstrap values (99–100%). The two studied species, although both belonging to the tribe Lamiinae of the subfamily Lamioideae, exhibit evolutionary development that indicates a close relationship, and their similar morphology (leaves and flowers) also supports this kinship.

The bootstrap support value is 99–100% on each branch after 1000 bootstrap replicates. The bar of scaled branch is 0.005. The accession numbers of the two new chloroplast genomes are given as PV802395.1 (*Lamium barbatum*) and PV802396.1 (*Leucas ciliata*), listed behind their respective Latin names (in a purple square frame). The species name and accession number of the other 17 species downloaded are *Lamium takeshimense* (MN240520.1) (Park et al. 2019), *Lamium album* (NC_036971.1) (Park et al. 2019), *Lamium amplexicaule* (MT473770.1) (Zhao et al. 2021), *Lamium galeobdolon* (NC_036972.1) (Park et al. 2019), *Leucas mollissima* (OR565912.1), *Thymus mongolicus* (NC_046520.1) (Huaizhu et al. 2020), *Mentha canadensis* (NC_044082.1) (Huaizhu et al. 2019), *Lycopus lucidus* (PV558881.1) (Wang et al. 2021), *Rosmarinus officinalis* (NC_027259.1) (Wang et al. 2021), *Lagopsis supina* (NC_058246.1) (Bendiksby et al. 2011), *Phlomis fruticosa* (NC_065868.1) (Zhao et al. 2021), *Phlomoides rotata* (NC_065733.1) (Bendiksby et al. 2011), *Galeopsis bifida* (NC_071846.1) (Zhao et al. 2021), *Anisomeles indica* (NC_046781.1) (Zhao et al. 2021), *Salvia przewalskii* (NC_041091.1) (Du et al. 2019), *Salvia miltiorrhiza* (NC_020431.1) (Du et al. 2019), and *Lithospermum erythrorhizon* (NC_053783.1) (Park et al. 2020) selected as the outgroup.

## Discussion and conclusions

This study presents the first complete sequencing and analysis of the chloroplast genomes of *Lamium barbatum* and *Leucas ciliata*, two Lamioideae species from different genera with distinct morphological features. Phylogenetic analysis confirmed that *Lamium barbatum* is closely related to *Lamium tenuifolium* and *Lamium album*, consistent with previous findings (Park et al. 2019). Importantly, we identified a previously unknown close relationship between the two *Leucas* species. Additionally, five species from diverse Lamiaceae genera (*Lagopsis supina*, *Phlomis fruticosa*, *Phlomoides rotata*, *Galeopsis bifida*, and *Anisomeles indica*) were found to be genetically related to the studied species within a single branch. The SSR sequences identified here offer effective tools for plant variety identification, seed purity assessment, genetic relationship analysis, and molecular marker development. Complex SSR fragments, in particular, may serve as valuable genetic markers to accelerate the selection of superior varieties. The observed differences in morphology, chloroplast genome content, SSR sequences, and genetic relationships between these two species provide important resources for medicinal plant authentication, evolutionary studies, and germplasm selection.

## Supplementary Material

Figure S4.png

Figure S3.jpg

Figure S1.png

Table S.xlsx

Figure S2.jpg

## Data Availability

The genome sequences supporting the findings of this study are openly available in GenBank at NCBI (https://www.ncbi.nlm.nih.gov/) under accession numbers PV802395.1 and PV802396.1. The associated BioProject, Bio-Sample, and SRA numbers are PRJNA1276693, SAMN49081836 and SAMN49081837, and SRR33980865 and SRR33980864, respectively.

## References

[CIT0001] Bendiksby M, Thorbek L, Scheen AC, Lindqvist C, Ryding O. 2011. An updated phylogeny and classification of Lamiaceae subfamily Lamioideae. Taxon. 60(2):471–484. 10.1002/tax.602015

[CIT0002] Cantino PD, Sanders RW. 1986. Subfamilial classification of Labiatae. Syst Bot. 11(1):163–185. 10.2307/2418955

[CIT0003] Chang M, Chen X, Chen S, Ma L. 2014. Analysis of the diversity and geographical pattern of wild Lamiaceae species in China. Subtrop Plant Sci. 53(3):234–242.

[CIT0004] Du Q et al. 2022. Complete chloroplast genomes of two medicinal *Swertia* species: the comparative evolutionary analysis of *Swertia* genus in the Gentianaceae family. Planta. 256(4):73. 10.1007/s00425-022-03987-z 36083348

[CIT0005] Du Y, Wang YY, Xiang CL, Yang MQ. 2019. Characterization of the complete chloroplast genome of *Salvia przewalskii* Maxim. (Lamiaceae), a substitute for Dan-Shen *Salvia miltiorrhiza* Bunge. Mitochondrial DNA Part B. 4(1):981–982. 10.1080/23802359.2019.1581107

[CIT0006] Gomez-Casati DF, Barchiesi J, Busi MV. 2022. Mitochondria and chloroplasts function in microalgae energy production. PeerJ. 10:e14576. 10.7717/peerj.14576 36545385 PMC9762248

[CIT0007] Gui J-S, Wei Q-H, Yang S-D. 1991. Discussion on Leucas varieties in Yunnan. J Yunnan Coll Tradit Chin Med. 2:23–25. 10.19288/j.cnki.issn.1000-2723.1991.02.013

[CIT0008] Guzman E, Molina J. 2018. The predictive utility of the plant phylogeny in identifying sources of cardiovascular drugs. Pharm Biol. 56(1):154–164. 10.1080/13880209.2018.1444642 29486635 PMC6130559

[CIT0010] Huaizhu L et al. 2019. The complete chloroplast genome sequence of *Mentha canadensis* (Labiatae), a traditional Chinese herbal medicine. Mitochondrial DNA B Resour. 5(1):55–56. 10.1080/23802359.2019.1687031 33366419 PMC7721016

[CIT0009] Huaizhu L et al. 2020. The complete chloroplast genome sequence of *Thymus mongolicus* (Labiatae), a special spice plant. Mitochondrial DNA B Resour. 5(3):2597–2598. 10.1080/23802359.2020.1778573 33457873 PMC7782360

[CIT0015] Huang L, Yu H, Wang Z, Xu W. 2024. CPStools: a package for analyzing chloroplast genome sequences. iMetaOmics. 1(2):e25. 10.1002/imo2.25 PMC12806284 41676121

[CIT0011] Jin JJ et al. 2020. GetOrganelle: a fast and versatile toolkit for accurate de novo assembly of organelle genomes. Genome Biol. 21(1):241. 10.1186/s13059-020-02154-5 32912315 PMC7488116

[CIT0012] Kuang K et al. 2024. Research on phenolic acid components and their anti-inflammatory activity in *Leucas ciliata*. Chin J Tradit Chin Med. 49(11):2940–2946. 10.19540/j.cnki.cjcmm.20240218.201 39041153

[CIT0013] Langmead B, Salzberg SL. 2012. Fast gapped-read alignment with Bowtie 2. Nat Methods. 9(4):357–359. 10.1038/nmeth.1923 22388286 PMC3322381

[CIT0014] Li B et al. 2016. A large-scale chloroplast phylogeny of the Lamiaceae sheds new light on its subfamilial classification. Sci Rep. 6(1):34343. 10.1038/srep34343 27748362 PMC5066227

[CIT0016] Liu S et al. 2023. CPGView: a package for visualizing detailed chloroplast genome structures. Mol Ecol Resour. 23(3):694–704. 10.1111/1755-0998.13729 36587992

[CIT0017] Park I, Yang S, Song JH, Moon BC. 2020. Dissection for floral micromorphology and plastid genome of valuable medicinal borages *Arnebia* and *Lithospermum* (Boraginaceae). Front Plant Sci. 11:606463. 10.3389/fpls.2020.606463 33343605 PMC7746654

[CIT0018] Park KT, Shin J, Park S. 2019. Complete chloroplast genome of *Lamium takesimese* Nakai (Lamiaceae): an endemic species in South Korea. Mitochondrial DNA B Resour. 4(2):3216–3217. 10.1080/23802359.2019.1667899 33365926 PMC7706697

[CIT0019] Tjendana Tjhin V et al. 2024. Baseline data collections of lipopolysaccharide content in 414 herbal extracts and its role in innate immune activation. Sci Rep. 14(1):15394. 10.1038/s41598-024-66081-2 38965275 PMC11224407

[CIT0020] Wang J et al. 2024. Plant organellar genomes: much done, much more to do. Trends Plant Sci. 29(7):754–769. 10.1016/j.tplants.2023.12.014 38220520

[CIT0021] Wang Q, Luo S, Xu Y. 2012. Study on the chemical composition of *Leucas ciliata*. Chin Herb Med. 43(1):27–31.

[CIT0022] Wang Y, Wang H, Zhou B, Yue Z. 2021. The complete chloroplast genomes of *Lycopus lucidus* and *Agastache rugosa*, two herbal species in tribe *Mentheae* of Lamiaceae family. Mitochondrial DNA B Resour. 6(1):89–90. 10.1080/23802359.2020.1847617 33521278 PMC7819123

[CIT0023] Wickham H. 2016. ggplot2: elegant graphics for data analysis. Springer-Verlag.

[CIT0024] Xiang CY et al. 2023. Using PhyloSuite for molecular phylogeny and tree-based analyses. Imeta. 2(1):e87. 10.1002/imt2.87 38868339 PMC10989932

[CIT0025] Yuan L et al. 2024. Comparative chloroplast genomes study of five officinal *Ardisia* species: unraveling interspecific diversity and evolutionary insights in *Ardisia*. Gene. 912:148349. 10.1016/j.gene.2024.148349 38460806

[CIT0026] Zhang Y, Tian L, Lu C. 2023. Chloroplast gene expression: recent advances and perspectives. Plant Commun. 4(5):100611. 10.1016/j.xplc.2023.100611 37147800 PMC10504595

[CIT0027] Zhao F et al. 2021. An updated tribal classification of Lamiaceae based on plastome phylogenomics. BMC Biol. 19(1):2. 10.1186/s12915-020-00931-z 33419433 PMC7796571

