# Supplementary figures and images for: Complete chloroplast genomes analysis of Lamium barbatum and Leucas ciliata (Lamiaceae)
Source: Mitochondrial DNA B Resour. 2025 Nov 9;10(12):1138–42. doi: 10.1080/23802359.2025.2582528 (PMC12604122; doi:10.1080/23802359.2025.2582528)

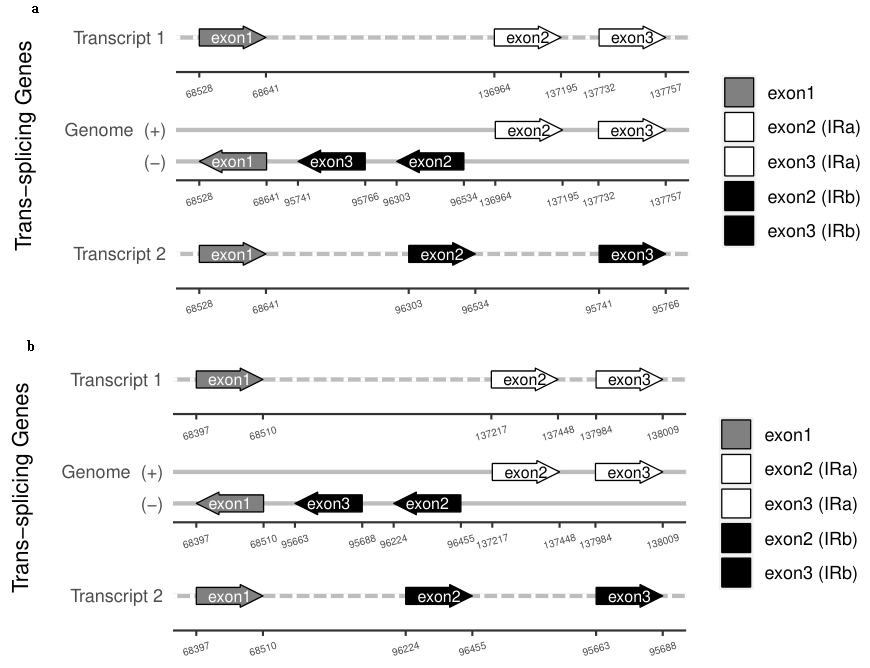

Supplement: Figure S4.png [file TMDN_A_2582528_SM7641.png]

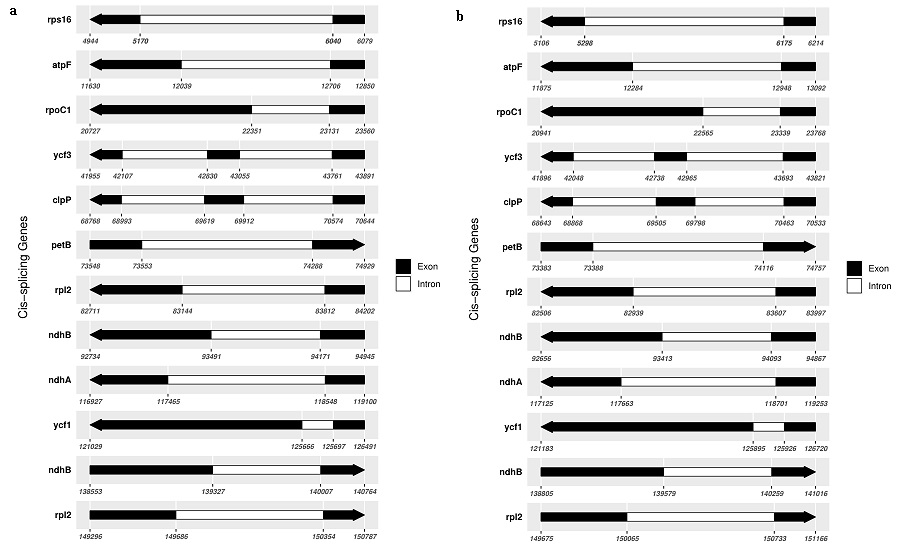

Supplement: Figure S3.jpg [file TMDN_A_2582528_SM7640.jpg]

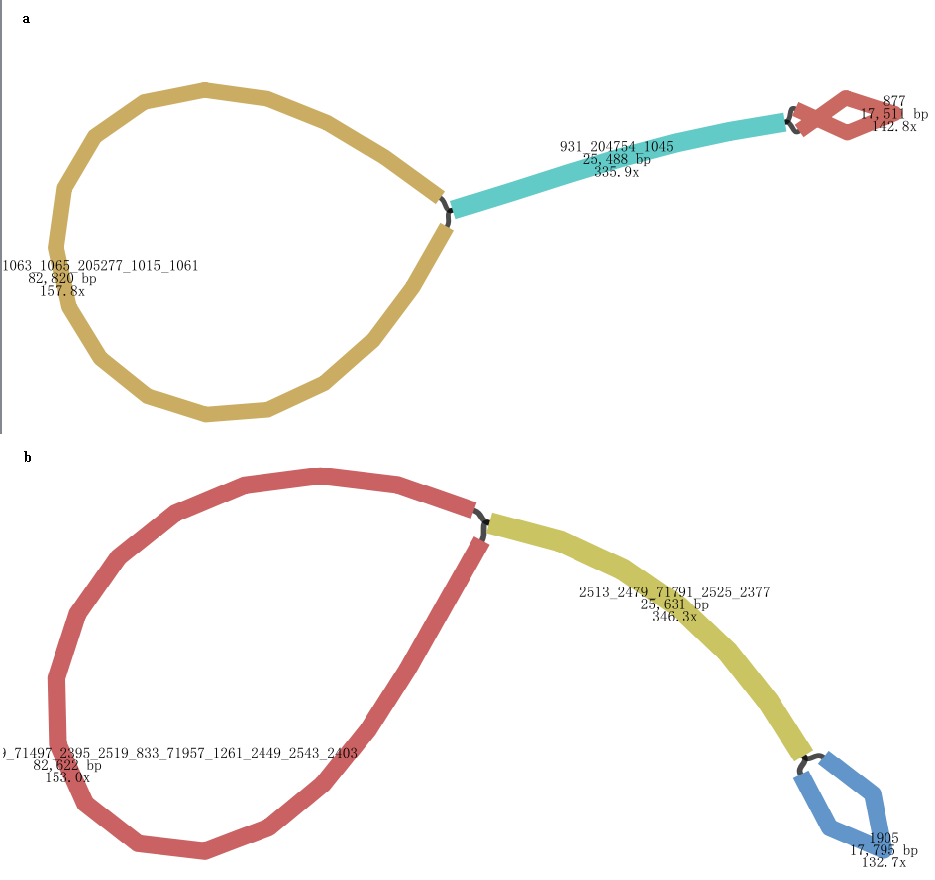

Supplement: Figure S1.png [file TMDN_A_2582528_SM7639.png]

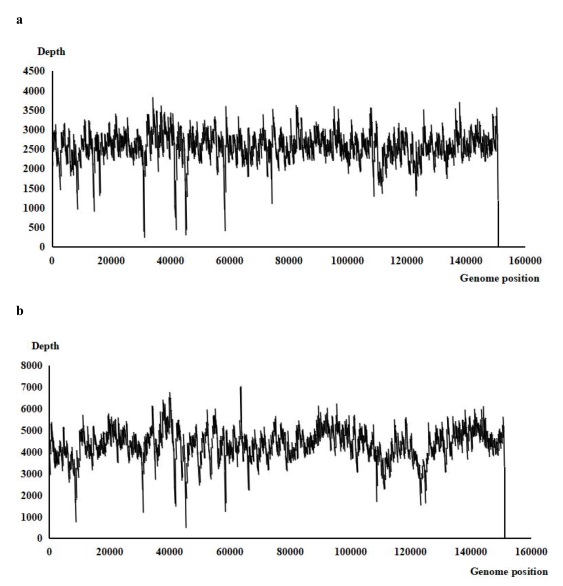

Supplement: Figure S2.jpg [file TMDN_A_2582528_SM7637.jpg]
